# Bilateral Sixth Nerve Palsy: A Rare Presentation of Primary Hypophysitis

**DOI:** 10.7759/cureus.58850

**Published:** 2024-04-23

**Authors:** Josue D Pagoada-Torres, Rodolfo Villalobos-Díaz, Luz M Pineda-Centeno, Luis Pesci-Eguia, Thamar Gomez-Villegas, Hector Rivera-Montes, Lesly A Portocarrero-Ortiz

**Affiliations:** 1 Endocrinology, Instituto Nacional de Neurología y Neurocirugía "Manuel Velasco Suárez", Mexico City, MEX; 2 Neurosurgery, Instituto Nacional de Neurología y Neurocirugía "Manuel Velasco Suárez", Mexico City, MEX; 3 Neurology, Instituto Nacional de Neurología y Neurocirugía "Manuel Velasco Suárez", Mexico City, MEX; 4 Ophthalmology, Instituto Nacional de Neurología y Neurocirugía "Manuel Velasco Suárez", Mexico City, MEX; 5 Neuroendocrinology, Instituto Nacional de Neurología y Neurocirugía "Manuel Velasco Suárez", Mexico City, MEX

**Keywords:** methylprednisolone, hypophysis, hypopituitarism, nerve palsy, hypophysitis

## Abstract

Cranial nerve palsy is common in pituitary disease and depends on the extension of the lesion into the cavernous sinuses. Bilateral cranial nerve palsy was described in pituitary adenomas with apoplexy and in only one case in hypophysitis. We present a case of a 32-year-old female manifesting with headache, diplopia, bilateral sixth nerve palsy, and hypopituitarism. Magnetic resonance imaging (MRI) revealed symmetric expansion of the pituitary gland, with bilateral cavernous sinus invasion and thickening of the pituitary stalk. Hypophysitis was suspected, and after treatment with IV methylprednisolone boluses, a decrease in the pituitary lesion was observed, with complete remission of sixth nerve palsy in the right eye and partial improvement in the left eye. In this case, we report an infrequent form of presentation of hypophysitis, and highlight that steroids are the first line of treatment.

## Introduction

Cranial nerve palsy can occur in pituitary disease depending on the extension of the lesion into the cavernous sinuses. It was described with pituitary adenomas in 2.4-32% of cases [1-4] and may be associated with pituitary apoplexy and infiltrative pituitary disease such as hypophysitis. 

Hypophysitis is a rare condition, with an overall incidence of 1 case per 7-9 million individuals. It is characterized by autoimmune inflammation of the pituitary gland and stalk, and its etiology can be classified into primary and secondary causes. The most frequent clinical manifestations of hypophysitis are hormonal deficiencies (66-97%), headache (61%), and visual symptoms (40%) [5]. As mentioned above, cranial nerve palsy is an unusual manifestation caused by lateral extension of the lesion into one or both cavernous sinuses and occasionally results in total or partial occlusion of the cavernous portions of the internal carotid arteries [2]. Cranial nerve palsy is an unusual manifestation of hypophysitis. Hunn et al. reported nerve palsy in 26.8% of cases. and Beressi et al. reported unilateral abducens nerve compromise in hypophysitis in 4.2-7.3% of cases [6,7]. Bilateral nerve palsy of the abducens was reported in one patient with hypophysitis [8]. In this case, we illustrate a rare manifestation of hypophysitis and its response to glucocorticoid treatment. 

## Case presentation

A 32-year-old Mexican woman with no recent history of childbirth, last menstrual period two months before evaluation, and no other relevant medical history complained of subacute onset of a stabbing forehead headache of moderate intensity associated with nausea without vomiting. The patient decided to consult a physician who prescribed treatment with ibuprofen as the headache progressively worsened within a week, evolving into an incapacitating headache. She decided to visit our emergency department as the headache persisted with severe intensity, and three months after the clinical onset, she developed diplopia and galactorrhea during breast manipulation. At the first physical examination, a bilateral sixth nerve palsy was revealed (horizontal binocular diplopia, which worsened on attempted abduction, esotropia, 45 prismatic diopters, and -2 abduction limitation in both eyes), without abnormalities in visual acuity (20/20 bilateral) or campimetry (confrontation campimetry). The hormonal profile showed secondary hypothyroidism, secondary adrenal insufficiency, and hypogonadotropic hypogonadism (Table 1). As the pregnancy test and COVID-19 serology were negative, levothyroxine and prednisone were prescribed.

**Table 1:** Summary of patient’s laboratory at diagnosis and follow-up ACTH: Adrenocorticotropic hormone, FSH: follicle-stimulating hormone, FT4: free thyroxine hormone, IGF-1: Insulin-like growth factor 1, LH: Luteinizing, TSH: thyroid-stimulating hormone

	At diagnosis	1 month after treatment	6 months after treatment	12 months after treatment	Normal reference range
TSH (µIU/mL)	0.081	0.015	0.336	0.167	0.47-4.68
FT4 (pmol/L)	5.7	14.1	15	16.9	10.0-28.2
FSH (mUI/mL)	1.4	1.6	4.6	6.25	1.55-9.74
LH (mUI/mL)	0.20	0.2	4.7	9.36	1.9-9.2
Estradiol (pg/mL)	9.372	10.6	33.93	40.3	26.6-161
IGF-1 (ng/mL)	242.2	182	125.6	61	59-279
Prolactin (ng/mL)	70.9	120.6	26.2	15.1	3.3-26.7
Cortisol (µg/dL)	0.8	0.4	8.7	14.7	4.46-22.7
ACTH (pg/mL)	1.6	1.6	24.9	37.2	4.7 - 48.8

**Figure 1:**
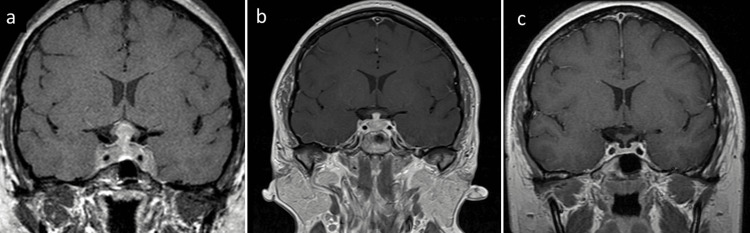
MRI findings at diagnosis and follow-up Magnetic resonance imaging of the pituitary gland (coronal section, T1 weighted, post-gadolinium). (a) Symmetric enlargement of the pituitary gland with bilateral extension to the cavernous sinuses (predominantly on the left, and thickening of the pituitary stalk (10.9 mm) at diagnosis (b) significant reduction in the size of the pituitary gland and pituitary stalk (4.05 mm) with less extension to the cavernous sinuses during follow-up, one month after treatment; (c) normal size of the pituitary gland and pituitary stalk (1.44 mm) fifteen months after treatment.

**Figure 2:**
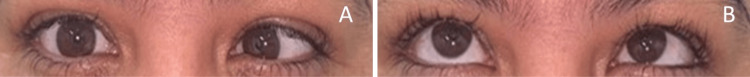
Improvement in sixth cranial nerve palsy One month after treatment, remission of sixth cranial nerve palsy in the right eye and improvement in the left eye. A: primary position; B: elevation.

Magnetic resonance imaging (MRI) revealed symmetric expansion of the pituitary gland, with bilateral cavernous sinus invasion and thickening of the pituitary stalk (Figure 1). The patient was evaluated by neuroendocrinology, and infundibulohypophysitis was considered. Clinical signs or symptoms related to systemic inflammatory disease were not found, and there was no history of exposure to checkpoint immune inhibitors, leading to a suspicion for primary hypophysitis. The patient was treated with IV methylprednisolone boluses and oral prednisone. In the follow-up examination, resolution of the sixth nerve palsy in the right eye and partial improvement of the left eye were observed (esotropia and -1 abduction limitation in the left eye) (Figure 2).

The prednisone dose was tapered after three months of treatment until withdrawal, without relapse during fifteen months of follow-up, and cortisol replacement was not needed (matutine serum cortisol 14.7 μg/dl). The patient continued treatment with levothyroxine, and replacement with estrogen and progesterone was initiated for hypogonadotropic hypogonadism. Prolactin levels were within the normal range (15.1 ng/ml). The last MRI (15 months after diagnosis) showed a significant reduction in the size of the pituitary lesion and pituitary stalk with less extension to the cavernous sinuses.

## Discussion

The incidence of ocular palsy occurring with pituitary tumors is between 2.4% and 32% [1-4]. The third cranial nerve was the most frequently affected, followed by the sixth, fourth, or fifth cranial nerve palsy, in that order in previous reports [1,9], and the sixth and fourth cranial nerves in recent data [10]. The prevalence increases to 47-70% in the presence of pituitary apoplexy, with the third nerve remaining the most frequently affected [11,12]

In hypophysitis, visual disturbance occurs in 40% of cases, and diplopia is an unusual manifestation. In a systematic review of 82 cases of idiopathic granulomatous hypophysitis by Hunn et al. [6], cranial nerve palsy was found in 26.8% of cases, including 6 cases (7.3%) of abducens nerve compromise. In another systematic review of 145 cases of lymphocytic hypophysitis by Beressi et al. [7], there were seven cases (4.1%) of diplopia and sixth cranial nerve palsy.

We report the case of a 32-year-old woman with bilateral sixth cranial nerve palsy secondary to infundibulohypophysitis. The patient had a favorable response to glucocorticoid therapy with subsequent improvement of motor ocular impairment and persistence of hypopituitarism. We excluded pituitary adenoma and apoplexy on the basis of clinical characteristics, imaging data, and treatment response. The evolution of the symptoms was subacute, without thunderclap headache, MRI with the presence of symmetrical pituitary lesion with thickening of the pituitary stalk and absence of areas suggestive of infarction or hemorrhaging, and reduction in the size of the pituitary lesion and pituitary stalk after treatment with steroids. In this case, as it was not possible to establish the etiology of hypophysitis, we decided not to perform a biopsy because of a favorable response to treatment and the risk of hospitalization in the context of a critical point of the pandemic due to COVID-19.

Gadolinium-enhanced pituitary MRI, with thin slices (2-3 mm or thinner) and a half dose of gadolinium (0.05 mmol/ kg), is the neuroimaging study of choice [13]. MRI features suggestive of hypophysitis include an enlarged triangular- or dumbbell-shaped gland, pituitary stalk thickening, generally homogeneous contrast enhancement, and absence of a bright posterior pituitary spot on T1w images, particularly in patients presenting with vasopressin deficiency (diabetes insipidus). This extension may be suprasellar or parasellar into the cavernous sinuses [5]. The characterization of a sellar mass generally results in a wide differential, with no single radiologic feature that can differentiate between different sellar pathologies, such as adenomas [14]. There is no radiologic pattern to differentiate between the most frequent types of primary hypophysitis, lymphocytic hypophysitis, granulomatous hypophysitis, and IgG4-related diseases that present with similar radiologic findings. Xanthomatous hypophysitis presents as cystic sellar masses on MRI. This feature can help distinguish it from other types of hypophysitis. To aid the distinction between pituitary adenoma and hypophysitis, a neuroradiological scoring system has been proposed. However, its discriminatory value has not been compared with other diagnostic tools [15]. 

The definitive diagnosis of hypophysitis is made through histopathological examination of a biopsy specimen; however, this is an invasive procedure associated with morbidity and, in some cases, mortality and may not be necessary unless the management outcome of the biopsy outweighs its risks.

In the absence of symptoms of systemic inflammatory disease, primary hypophysitis was suspected. Post-COVID-19 lymphocytic hypophysitis has been described; however, in this case, as the PCR (nasal swab) for COVID-19 was negative, treatment with glucocorticoids and hormonal replacement was indicated, and definitive etiology was not achieved because a biopsy was not performed. 

Hormonal deficiencies are common in hypophysitis (66-97%) making the evaluation of anterior pituitary hormones mandatory. In lymphocytic hypophysitis, ACTH, gonadotropin, TSH, and GH deficiencies are described in this sequence. Vasopressin deficiency (diabetes insipidus) affects up to 50% of patients but is unusual in immune checkpoint inhibitor-induced hypophysitis cases [5, 14]. Approximately 25-50% of patients treated with glucocorticoids show improved pituitary function. In pituitary apoplexy, bilateral oculomotor nerve palsy has been described in some reports [16-18]. At the time of writing this paper, there is only one report of bilateral sixth nerve palsy in the setting of hypophysitis [6].

Although medical therapy with glucocorticoids remains the first-line treatment for hypophysitis, up to 38% of cases may recur after glucocorticoid discontinuation. In these cases, other immunosuppressive drugs, such as azathioprine, methotrexate, infliximab, cyclosporine, and rituximab, can be considered. Surgical intervention is reserved for diagnostic purposes, through a pituitary biopsy, or in a subset of patients unresponsive to medical therapy [5,19].

In this case, management was initiated with intravenous glucocorticoids followed by high-dose oral glucocorticoids, and the follow-up steroid was tapered until withdrawal without relapses at the time of writing this paper.

## Conclusions

In the setting of a patient with headache, diplopia, visual field alterations, and cranial nerve palsy in pituitary disease should be considered. Cranial nerve palsy in pituitary disease is secondary to the extension of the lesion into the cavernous sinuses. The third, sixth, and fourth cranial nerves are most frequently affected. Bilateral cranial nerve palsy is uncommon in pituitary disease. This presentation was seen more frequently in patients with pituitary adenomas in the context of pituitary apoplexy. In the case of hypophysitis, primary and secondary etiologies must be investigated; lymphocytic hypophysitis is the most common type. MRI with gadolinium is the imaging study of choice. However, there is no radiological pattern to distinguish the most frequent hypophysitis. Anterior pituitary hormone and vasopressin deficiencies should be investigated. This case highlights that the first-line treatment for hypophysitis is steroids and hormone replacement, even in rare manifestations.
